# Monoterpenes of *Salvia leucophylla*

**DOI:** 10.2174/157340712799828205

**Published:** 2012-01

**Authors:** Atsushi Sakai, Hiroko Yoshimura

**Affiliations:** 1 Department of Biological Sciences, Faculty of Science, Nara Women’s University, Kitauoya-nishi-machi, Nara, 630-8506, Japan; 2 Department of Biological Sciences, Graduate School of Humanities and Sciences, Nara Women’s University, Nara, 630-8506 Japan

**Keywords:** Chemical behavior, cuticle layer, mode of action, monoterpenes, Salvia phenomenon.

## Abstract

The “ *Salvia* phenomenon” is one of the most famous examples of allelopathic interaction between higher plants. The *Salvia *thickets are surrounded by zones of bare soil (“bare zone”, 1-3 m in width), which merge into areas of inhibited grassland (“zone of inhibition”) and finally undisturbed grassland at a distance of 3-9 m. This characteristic vegetation pattern was attributed to monoterpenes, especially 1,8-cineole and camphor, which volatilized from *S. leucophylla *leaves, got adsorbed in the soil around the *Salvia *thickets, and inhibited germination and seedling growth of annual herbs. Initially, continuity of hydrophobic environment (clay soil particles – cuticular waxes on the seed/seedling surfaces – plasmodesmata - plasma membrane) was regarded to be important for the lipophilic compounds to enter the target cells. However, monoterpenes can reach the target cells via aqueous route as well. Because monoterpenes produced by *S. leucophylla *all induce similar symptoms in the seedlings of target plants, their mode of action appears to be essentially common. They exert various deteriorating effects on the cells of target plants, which might be totally explained if the primary point of action resides in mitochondrial function (respiratory ATP synthesis) and/or generation of reactive oxygen species. In contrast to the previous belief that cuticular waxes act as the pathway of lipophilic monoterpene to enter the site of action or reservoir of the inhibitors, they may act as “adsorptive barrier” to prevent the entering of monoterpenes inside the cell wall.

## INTRODUCTION

*Salvia leucophylla* (purple sage, gray sage, or wild Carifornia sage) is an aromatic shrub dominant in the coastal sage scrub in southern California. C.H. Muller *et al*. [1] reported a striking pattern of distribution of herbs around the thickets of *S. leucophylla* in the Santa Yenz Valley, Santa Barbara County, California. Usually, annual grasses and forbs are excluded from interiors of the shrub thickets. The *Salvia* thickets are surrounded by zones of bare soil (“bare zone”, 1-3 m in width), which merge into areas of inhibited grassland (“zone of inhibition”) and finally undisturbed grassland at a distance of 3-9 m (Fig. (**1**)). As a result of intensive studies, C.H. Muller and his colleagues proposed a hypothesis as follows [2]:

- *S. leucophylla* leaves produce and release monoterpenes, mainly 1,8-cineole and camphor, into the atmosphere.
- The volatilized monoterpenes are adsorbed in the soil particles around the *Salvia* thickets and retained there for at least several months.
- The soil-bound monoterpenes inhibit the seed germination and seedling growth of annual grasses and forbs, contributing to the above-mentioned characteristic “bare zones”.

This “*Salvia* phenomenon” is one of the most famous examples of allelopathic interaction between plants reported to date, although more detailed and quantitative analyses on every step of the proposed process, as well as a critical evaluation of the importance of monoterpenes in establishing the characteristic distribution patterning of grassland species around the *Salvia* thickets, seem to be necessary for its correct understanding. In this review, we first describe the above-mentioned hypothesis in some detail, and then examine the validity of each step of the proposed processes, with special emphasis on the incorporation into target plants and mode of action of monoterpenes*. *

## SALVIA LEUCOPHYLLA

*Salvia leucophylla* (Family Lamiaceae) is an evergreen, aromatic shrub dominant in the coastal sage scrub in southern California. It grows up to about 120 cm in height and width. The stems are woody below, herbaceous above and bear opposite, short-petiolate, lanceolate to oblong-lanceolate leaves with white tomentose lower surfaces [3]. Its specific name (*leucophylla*) means the light-gray color of the leaves. The common names come from its flower color (purple sage), leaf color (gray sage), and habitat (wild Carifornia sage), respectively.

## *SALVIA* PHENOMENON: SPATIAL DISTRIBUTION OF ANNUAL GRASSLAND SPECIES IN AND AROUND *SALVIA* THICKETS

Under the Mediterranean climate of coastal southern California, *S. leucophylla*, together with *Artemisia californica* (California sagebrush, Family Asteraceae) forms a soft chaparral vegetation adjacent to natural grassland, which consists of a number of annual species (e.g., *Avena fatua*, *Bromus mollis*, *Festuca megalura*, and *Erodium cicutarium*) and a few perennial species (e.g., *Stipa lepida* and *Poa scabrella*). Usually, grasses and forbs are excluded from interiors of the shrub thickets, although there are exceptional cases in which annual herbs grow luxuriantly beneath *Salvia* thickets [4]. The zones of contact between *Salvia* thickets and grassland characteristically exhibit “bare zones” extending 1 to 2 meters beyond the *Salvia* crowns. The soil within this zone is completely barren of herbs or exhibits only sparsely scattered and stunted seedlings of several selective annual species (*Erodium*, *Festuca*, and *Bromus mollis*); however, these seedlings cannot grow to maturity. The 3rd to 6th meters beyond the shrub bear dense but stunted herbage of the several selective annuals; they can mature and gain a few seeds. More than 6 to 10 meters beyond the shrubs, this inhibited vegetation gradually merges with normal, uninhibited grassland where various herbs, including *A. fatua* that cannot be found in the vicinity of *Salvia* thickets, exist. The “inhibition zone” extends far beyond the reach of the roots and/or crowns of the shrubs, and its size is approximately equal between uphill- and downhill-sides.

In addition to the “inhibition” of annuals outside the thickets, a sign for self-deterioration inside the stands can also be found in large and old stands of the shrubs**[4]. Individual shrubs in smaller and younger thickets are vigorous and forming dense crowns. In contrast, each shrub in the interior of larger and older stands forms a small crown with few leaves, and there are many areas of bare soil between the shrubs. Moreover, the shrub seedlings tend to be established only in the “bare zone” and “inhibited area”; in spite of the available space and enough light, the shrub seedlings are absent from the interior of such large, old stands.

## HYPOTHESIS ABOUT “*SALVIA* PHENOMENON” 

### I Inhibition of Seedling Growth by Volatile Materials Emanating from *Salvia* Leaves

Mineral and physical properties of soils showed no significant differences across *Salvia*-grassland contact (i.e., among interior of *Salvia* thicket, bare zone, inhibited area, and grassland), indicating that the characteristic vegetation around *Salvia* thickets could not be correlated with any edaphic factors [4]. The observation that the “inhibited grass” could not grow vigorously even if mineral nutrients was supplied as manure deposit demonstrated that the depletion of nutrients was not the mechanism of the growth inhibition around *Salvia* thickets. The stunting or inhibition of herb growth occurred even during periods of favorable soil moisture, indicating that growth inhibition around *Salvia* thickets could not be ascribed to moisture depletion. Finally, the stunting of the herbs was observed to occur several meters beyond the reach of canopies and root systems of the shrubs. Thus, it seemed unlikely that competition for resources (such as light, water, and minerals) caused the growth inhibition around the shrub thickets.

Another possible explanation was that animal activities might be involved in the formation of the bare - and inhibited-zones around *S. leucophylla*. Long-term observations of the marked individual seedlings along permanent transects revealed that (i) there were few seedlings totally lost to grazing, (ii) there were many seedlings that remained permanently stunted even if they were protected from damage by grazing, and (iii) there were some inhibited zones with no evidence of grazing damage. Based on these observations, it was tentatively concluded that grazing by small animals alone could not explain the graded inhibition of herb growth around *Salvia* thickets.

While examining possible involvement of those various factors (such as resource limitation, competition for resources, environmental stress, and animal herbivory) in the formation of bare- and inhibited-zones around *Salvia* thickets, C.H. Muller and his colleagues regarded that allelopathy was an likely mechanism for the phenomenon, probably because of the highly aromatic nature of *S. leucophylla* [5]. Nonetheless, they first examined carefully the organ(s) that emanated the assumed inhibitor(s). Young and mature roots of *S. leucophylla*, whether macerated or not, did not inhibit the growth of test plant (cucumber) seedlings on filter paper to which *Salvia* root materials were in direct contact. Leachate from pots harboring *S. leucophylla* also failed to inhibit the germination and growth of cucumber [1]. The results of these experiments, together with the field observation that the inhibited zone spread equally to both uphill- and downhill-sides [4], ruled out the possibilities that (i) the roots released the hypothetical inhibitor(s) by exudation, (ii) the whole plants released the hypothetical inhibitor(s) by leaching, and (iii) the hypothetical inhibitor(s) had water-soluble nature. 

In contrast to the case with root materials, crushed leaves of *S. leucophylla* were inhibitory to seed germination and seedling growth of cucumber when they were assayed in a similar way, suggesting localization of toxic material(s) in the leaves. Because of the highly aromatic nature of *S. lucophylla* and the field observation that inhibition zone extended several meters beyond the reach of *Salvia* branches, volatile nature of the toxic material(s) was suspected. Muller and colleagues [1, 4] developed a new bioassay system to test the atmospheric transfer of the volatile toxins from *Salvia* leaves (Fig. (**2**)), and found that volatile materials emanating from crushed leaves of *S. leucophylla* could severely inhibited root growth of seedlings of not only cucumber (a model test plant) but also *A. fatua* (one of the herbs efficiently excluded from the vicinity of *S. leucophylla* stands in the field), in a dose-dependent manner. 

### II Terpenes Released from *Salvia* Leaves

W.H. Muller and C.H. Muller [6] found camphor, 1,8-cineole, dipentene, camphene, α -pinene, and β -pinene in the ether extract of *S. leucophylla* leaves (Fig. (**3**)). However, dipentene was not mentioned in later reports (e.g., [4]). Among them, camphor appeared to be most abundant, 1,8-cineole (and dipentene), α -pinene and camphene moderately abundant, while β -pinene least abundant. When *S. leucophylla* leaves were macerated, all of these terpenes were released and detected in the atmosphere above the macerated leaves. Among them, a-pinene and camphene appeared most abundant, 1,8-cineole (and dipentene) moderately abundant, while b-pinene and camphor least abundant in the headspace of the macerated leaves. Later, two of the terpenes, 1,8-cineole and camphor, were detected from the air around greenhouse- or field-grown, intact *S. leucophylla* [7]. 

Monoterpenes detected in the *S. leucophylla* leaves were all inhibitory to root growth of cucumber seedlings, and 1,8-cineole and camphor were the most toxic among them [6]. Moreover, when chemical compositions and growth-inhibitory activities of the volatiles emanating from macerated leaves of three *Salvia* species (*S. leucophylla*, *S. apiana*, and *S. mellifera*) were examined, the quantities of the terpenes corresponded closely to the degree of inhibition of cucumber growth by each *Salvia* species [6]. These observations suggested that the growth inhibition of annual grassland species within and around *S. leucophylla* stands was due to the production and release of the volatile terpenes, especially camphor and 1,8-cineole, from its leaves.

### III Transport to Target Plants and Condensation of Terpenes

Under natural circumstances, terpenes must be evaporated from uninjured leaves and deposited upon the target seeds/seedlings. Moreover, the concentration of the terpenes in the atmosphere around *Salvia* plants was quite lower than the concentration, in the bioassay chambers, required to cause toxicity. Thus, there must be mechanisms to (i) deliver atmospheric terpenes to seeds/seedlings at/under the soil surface, and (ii) increase their concentrations at the site of action, if the terpenes were indeed involved in the growth inhibition around *Salvia* plants. Initially, it was hypothesized that the terpenes might be trapped during the precipitation of dew [1], because artificially precipitated dew taken from atmosphere among *Salvia* plants contained terpenes and showed growth-inhibitory activity. However, dew is formed during periods of chilling, when terpenes should be produced at only low rate. Then, it was proposed that the atmospheric terpenes were directly dissolved in the waxes of cutin of the seedlings of target plants [7], because terpenes were rapidly and efficiently dissolved in solid paraffin and the surface of plant body was covered with cuticular wax. This “direct adsorption model” appeared more realistic than the previous “dew model”, because terpenes would be deposited much more during warm season than during cooler, dew-producing season. However, the inhibition should have occured at the beginning of the growing season, because growth inhibition of annual grassland species became clear by the middle of the growing season. Finally, C.H. Muller and del Moral [2] found that dry soil could rapidly and efficiently adsorb terpenes from atmosphere. Fresh soil treated with volatiles from macerated *S. leucophylla* leaves became highly inhibitory to seedling growth, and the soil toxicity was retained for several months. Based on these results, it was suggested that terpenes, volatilized during hot season from *Salvia* leaves, were adsorbed by dry soil, remained there for several months, and inhibited the growth of seedlings of annual herbs in the beginning of the next growing season.

### IV Penetration into Target Plants 

Based on the considerations that (i) terpene vapors are quickly dissolved in hard paraffin, (ii) the cuticular layers of the leaves extend into the mesophyll and cover the surfaces of all cells exposed to an intercellular space within a leaf, (iii) plasmodesmata extend through the cell walls and make contact with those cuticular layers, and (iv) plasma membranes are composed of lipids, C.H. Muller and del Moral [2] proposed that the terpenes adsorbed to soil might be transported to the sites of inhibition in the cytoplasm through lipophilic environment (i.e., cuticular wax, plasmodesmata, and plasma membranes).

### V Mechanism of Inhibition

The mechanism how the terpenes inhibit seedling growth is not yet solved clearly. It has been demonstrated that exposure of test plants to the volatiles emanating from *S. leucophylla* leaves, or to vapors of 1,8-cineole and camphor, results in severe inhibition of root and hypocotyl growth [8]. A marked reduction in cell division, cell elongation, and lateral root initiation was observed in cucumber seedlings treated with those vapors [9]. Respiratory activity of the seedlings and excised organs was inhibited by exposure to those vapors [10]. Moreover, 1,8-cineole, one of the most abundant and inhibitory terpenes released from *S. leucophylla* leaves, reduced oxygen uptake by isolated mitochondria [11]. These observations suggest that mitochondrial respiration might represent a target point of inhibition by terpenes.

Microscopic observations of the seedlings exposed to terpenes demonstrated widespread systemic disturbances. Lipid globules accumulated within most cells, and excessive cutin deposition on the outer walls of epidermal cells was noted [9]. Electron microscopic examination confirmed accumulation of globules in the cytoplasm, drastic reduction in the number of intact organelles, and disruption of membranes surrounding organelles such as nuclei, mitochondria, and Golgi apparatus [12]. These disturbances were supposed to be involved in the inhibition of several annual grassland species around *Salvia* plants in the field.

## RE-EXAMINATION OF THE “*SALVIA* PHENOMENON” 

### I Ecological Relevance

As described in some detail above, this series of study started to explain the mechanism(s) to form bare- and inhibited-zones around *Salvia* thickets. Soon after the publish of the report to suggest allelopathic nature of the *Salvia* phenomenon [1], P.V. Wells [13] suggested that most of the bare zones represented cattle trails. C.H. Muller and W.H. Muller [14] immediately responded to this suggestion. They argued, based on the measurement of the distribution of cow droppings, that the cows went where grass was abundant and that they did not linger about shrub thickets. Thus, the “cattle trail explanation” appeared to be rejected, but possible involvement of animal activity was again suggested. Bartholomew [15] pointed out that shrub thickets might provide excellent cover for small animals such as rodents, rabbits, and birds, and demonstrated that (i) there was increased animal activity adjacent to shrub thickets and that (ii) annual grassland species could grow in the “bare zone” when animal activity was prevented with wire-mesh exclosures, suggesting that the animal activity was sufficient to produce the bare zone. C.H. Muller and del Moral [16] showed several instances suggesting that bare zones could develop in the absence of animal pressure. However, Bartholomew [17] immediately criticized that the points presented in that letter [16] were not the conclusive evidence for chemical inhibition, and stressed that “The extent of the relative contribution of chemical and animal inhibition to the formation and maintenance of the bare zones needs further investigation”. Bartholomew was invited to C.H. Muller’s lab for discussion, and the members who joined the discussion recognized the importance of animal activity in the formation and maintenance of “bare zones” [18]. 

Later, Halligan [19] carefully examined the case with bare zone around *Artemisia carifornica* (an aromatic, monoterpene-producing shrub native to California coastal vegetation like *S. leucophylla*). The results of the experiments with exclosures strongly suggested a central role for small mammals in causing the bare zones around *A. californica* stands. However, among annual native grassland species around *A. californica*, *Hypochoeris glabra* (smooth cat’s ear) and *Madia sativa *(coast tarweed) appeared to be still inhibited, whereas *Bromus diandrus* (rip-gut grass) got to grow well, when protected from grazing in the shrub zone. Moreover, bioassays to test toxicities of volatiles from *A. californica* leaves, artificial rain drip from boughs of *A. californica*, and soil from the shrub zones, all demonstrated that the former two species (*H. glabra* and *M. sativa*) were inhibited by materials derived from *A. californica* while the latter one (*B. diandrus*) was not, suggesting that some species were allelopathically kept away from *A. californica* thickets while others were not. In conclusion, both allelopathy and animal herbivory appear to play respective roles in forming the characteristic vegetation pattern. Halligan [19] stated that both C.H. Muller [4] and Bartholomew [15] was correct, but oversimplified the herb pattern and overrated the importance of only one factor. 

### II Site of Synthesis and Storage of Monoterpenes in *Salvia* *Leucophylla*

C.H. Muller *et al*. [1], based on bioassay using leaves, roots, and whole-plant leachates as potential donor of growth inhibitor(s), proposed leaf origin of the toxic material(s) of *S. leucophylla*. The pattern of herb inhibition around *Salvia* thickets strongly suggested volatile nature of the hypothetical growth inhibitor(s). Several monoterpenes, which were highly volatile and each inhibited seedling growth to various extents, were indeed detected in the leaves [6]. The monoterpenes that W.H. Muller and C.H. Muller found in the *S. leucophylla* leaves were camphor, 1,8-cineole (plus dipentene), camphene, a-pinene, and b-pinene (in the order of decreasing contents). More quantitative study on the terpene composition of *S. leucophylla* [20] revealed the presence of camphor (plus borneol, ca. 50%), 1,8-cineole (ca. 30%), a-pinene (ca.. 7%), camphene (ca. 5%), b-pinene (ca. 3%), and limonene (dipentene, ca. 2%). Terpenes are major constituents of essential oils. As revealed by detailed and highly sensitive analyses on the chemical composition of essential oils from several *Salvia* species (e.g., [21, 22]), each *Salvia* species contains various kinds of monoterpenes whose compositions varies from species to species. That camphor and 1,8-cineole represent major constituents of essential oils seems to be a characteristics of *Salvia* section *Audiberita* [23] to which *S. leucophylla* and several other California *Salvia*s belong [20, 22].

Monoterpenes are synthesized by monoterpene synthases. The vast diversity of monoterpenes within and among species is attributable to the large number of different terpene synthases and the nature of some terpene synthases that can produce multiple products [24]. At present, about 90 genes encoding monoterpene synthases are known [25]. Five genes encoding monoterpene synthases have been cloned from three *Salvia* species; bornyldiphosphate synthase (BOR), 1,8-cineole synthase (CIN), and sabinene synthase (SAB) from *S. officialis*, CIN from *S. fruticosa*, and SAB from *S. pomifera *[26-28], although cloning of monoterpene synthase genes from *S. leucophylla* has not yet been reported [25]. These *Salvia* CIN genes appeared to be expressed in leaves, while *Arabidopsis* CIN gene in roots [29] and *Nicotiana* CIN gene in flowers [25].

As far as we know, localization of monoterpene synthases in *S. leucophylla* leaves has not been examined in detail. However, glandular trichomes on the leaf surface seem most likely sites of production and storage of monoterpenes. Glandular trichomes are widely distributed over the aerial organs of family Lamiaceae, including genus *Salvia, *and are the primary secretory organs of these plants [30]. Glandular trichomes are characterized by the tumescent, globular appearance of the cuticle that has split from the walls of secretory cells as the subcuticular space gets filled with secretory product [31]. Both upper and lower epidermal cell layers of *S. leucophylla* leaves are covered with numerous glandular and non-glandular hairs; Youngken and Heaps Jr. [3] illustrated several types of glandular hairs (trichomes) with 1-6 celled head and 1-3 celled stalk. In common sage (*S. officinalis*), camphor is the major constituents of essential oil. Camphor content of *S. officinalis* leaves increased as the leaves expanded and the oil-accumulating peltate glandular hairs increased in number [32], suggesting that camphor was synthesized and accumulated most actively in glandular hairs of young sage leaves.

### III Release of Monoterpens from *Salvia* *leucophylla*

How are terpenes accumulating in (glandular hairs of) *Salvia* leaves released into the environment? Basic mechanism of monoterpene volatilization has been studied in black sage (*S. mellifera*), which, like *S. leucophylla*, is an aromatic shrub growing in coastal Southern California [33, 34]*.* The rate of monoterpene volatilization from leaves was directly proportional to leaf temperature, the same in both light and dark, and independent of stomatal opening. These observations suggested that the terpenes were volatilized from leaf surface, rather than from leaf interior, by means of strictly physical mechanism.

Thus, it is certain that terpenes are volatilized from intact *Salvia* leaves into the air via physical mechanism. However, other pathways of terpene release may also exist. Tyson *et al.* [33] estimated the rate of terpene volatilization from *S. mellifera* leaves under natural conditions (those of April, 1972 at a coastal site of Camp Pendleton, California) to be 1.33 mg m^-2^ d^-1^. Based on this estimation, together with coverage by *S. mellifera* of the vegetation around study site (45.7%) and leaf area index of *S. mellifera* stands (2.6), the amount of terpene released into atmosphere was calculated to be 3.1 kg terpene km^-2^. This value was quite insufficient to fulfill the high levels of atmospheric organics measured in the field [35]. According to Rasmussen and Went [35], high levels of organic matter in the air was observed immediately after fields were mowed, or during the periods of maximum leaf drop in deciduous forests. This observation suggested that high levels of volatiles were primarily attributable to the loss from decomposing, dead leaves. Tyson *et al.* [33] pointed out that the *Salvia* leaves contained large amount of potentially volatile materials (3.15 g m^-2^) and that leaf drop and high summer temperature occurred simultaneously in the coastal sage communities, and suggested large amount of terpenes might be released from senescent, falling leaves.

In addition to the volatilization from senescent/dead leaves, leaching from living or dead leaves is another possible pathway of terpene release. Fischer *et al.* [36], while studying allelopathic phenomenon in Florida scrub, reported that aqueous soaks of fresh leaves of false rosemary (*Conradina canescens, *Family Lamiaceae) included various kinds of monoterpenes, including 1,8-cineole and camphor. They also demonstrated that aqueous soaks of false rosemary leaves, and aqueous solutions saturated with monoterpenes that were detected in the aqueous soaks as well, showed strong phytotoxicity. These observations suggest that monoterpenes stored in glandular trichomes are rather easily washed out with water, and the monoterpenes have sufficiently high water-solubilities to cause strong inhibitory effects in aqueous solution. Leaching from living leaves and decaying litter by rainfall is assumed to be the primary mechanism by which monoterpenes are released from scrub perennials in Florida [37].

Thus, while volatilization from intact leaves might represent the major route of release from *S. leucophylla* during dry season, volatilization from senescent/decaying leaves and leaching by rainfall/dew formation might contribute to the release of monoterpenes to the environment during rainy season. In *Arabidopsis*, synthesis of 1,8-cineole in the roots and its immediate release into the rhizosphere has been proposed [29]. However, the operation of similar mechanism in *S. leucophylla *seems unlikely, as judged from the results of initial bioassay [1].

### IV Behavior of Monoterpenes in the Environment

In general, aqueous transport may be essential to effective allelopathy [36]. Because of their low molecular weight and nonpolar characters, monoterpenes have been classified as volatile and assumed to have negligible solubility in water. Thus, C.H. Muller and del Moral [2] proposed the pathway that monoterpenes emanating from *Salvia* leaves were transported to the soil, where germination and seedling growth of target plants were inhibited. They demonstrated that the soil, especially when it was in dry condition, could adsorb volatile terpenes from the atmosphere. The soil exposed to volatiles emanating from macerated *S. leucophylla* leaves exhibited phytotoxicity, and the phytotoxicity was retained for several months. These observations led to the hypothesis that the monoterpenes volatilized from *S. leucophylla* leaves into the air were adsorbed onto the soil particle and accumulated there. Later, Halligan [19] demonstrated the phytotoxicity of soil around *A. carifornica* (an aromatic, monoterpene-producing shrub), supporting the terpene-charged soil theory. However, he also stated that the toxic effects were strongest by the time of first rain and that the toxic effects disappeared entirely by early spring (cited as pers. com. in [18]), probably because the toxins were washed away by winter rains. Then, toxins accumulating during the previous growth season could hardly prevent the germination and growth of herbs around aromatic shrubs.

It is certain that terpenes volatilized from leaves of aromatic shrubs are adsorbed to soil and make the soil toxic to several plant species. However, detailed analyses on the seasonal changes of terpene content and phytotoxicity of the soils around the aromatic shrubs, and careful examination of their correlation with life histories of aromatic shrubs and their neighboring herbs, seem necessary to evaluate the ecological significance of the adsorption and preservation of terpenes in the soil. 

### V Incorporation into Target Plants

Because of the presumed insolubility of monoterpenes in water, C.H. Muller and del Moral [2] regarded the continuity of lipophilic environment to be important when considering the way through which monoterpenes entered the interior of target plants, and proposed the following pathway: lipophilic soil particles – cuticular wax – plasma membrane at plasmodesmata – protoplasm. In contrast to general belief, however, the monoterpenes exhibit considerable solubility in water [38]. Among them, monoterpene hydrocarbons exhibited relatively low solubility (<35 ppm), but oxygenated monoterpenes exhibited relatively high solubilities; 155-6990 ppm for ketones including camphor (ca. 550 ppm), and 183-1360 ppm for alchols including 1,8-cineole (ca. 330 ppm). Because many monoterpenes appears active well below their solubilities in water [39], their penetration into target plants will not require continuous lipophilic corridor. The observation that 1,8-cineole vapor can affect the tobacco protoplasts suspended in liquid culture medium [40], which is described below, also support the opinion that monoterpenes can enter the target cells in the absence of continuous lipophilic corridor. 

### VI Mode of Action

Monoterpenes are known to inhibit respiration and mitosis, deteriorate membrane integrity, affect cuticlar waxes, enhance transpiration via stomatal opening, cause lipid oxidation, and disrupt microtubules [41-46]. Nonetheless, the molecular mechanism for the allelopathic effects of monoterpenes is still obscure.

Volatiles from *S. leucophylla* leaves and monoterpenes therein inhibited germination and seedling growth of various plant species, such as cucumber (*Cucmis sativus*, [6]), wild oat (*Avena fatua*, [1]), maize (*Zea mays*, [47]), potherb mustard (*Brassica rapa* var. *nipposinica, *formerly referred to as *B. campestris*, [48, 49]), barnyardgrass (*Echinochola crusgalli*) and sicklepod (*Cassia obtusifolia*, [43]), and tobacco (*Nicotiana tabacum*, [40]). These reports cumulatively suggest that the monoterpenes are more or less toxic to vast majority of various plant species. We recently confirmed this by examining the effects of 1,8-cineole on total of seven plant species including both dicotyledons and monocotyledons, under nearly equal experimental condition similar to that reported by Koitabashi *et al.* [48] (Fig. (**2**)). The test plants used were; potherb mustard (*B. rapa* var. *nipposinica*), tobacco (*N. tabacum*), Arabidopsis (*Arabidopsis thaliana*), lettuce (*Lactuca sativa*), garlic chives (*Allium tuberosum*), rice (*Oryza sativa*), and Bermuda grass (*Cynodon dactylon*). 1,8-Cineole inhibited seedling growth of all of these plant species in a dose-dependent manner (Table **1**). 

The next question is that whether the mode of action of monoterpenes is different from molecule to molecule. Within the plants treated with monoterpenes, growth was inhibited in both underground- and aboveground-parts [8, 40, 43, 48, 49] and, in most cases, root growth appeared more sensitive than hypocotyl growth to inhibition by monoterpenes. The higher sensitivity of root growth to 1,8-cineole in seedlings of various plant species is also apparent in the results shown in Table **1**. Nishida *et al.* [49] examined the effects of five monoterpenes produced by *S. leucophylla* (camphor, 1,8-cineole, β -pinene, α -pinene, and camphene) on the growth of potherb mustard seedlings. The five monoterpenes all inhibited seedling growth and, in all cases, the root growth was more sensitive to monoterpenes than hypocotyl growth. With appropriate doses where root growth was lowered to 25% of the control level while hypocotyl growth was not inhibited, the five monoterpenes did not affect the cell size (both in the root cortex and in hypocotyl epidermis) and mitotic index in the shoot apical region, but severely lowered mitotic index and DNA synthetic activity in the root apical meristem. The same response to the five *Salvia* monoter-penes observed in potherb mustard seedlings suggests that the mode of action of monoterpenes is essentially common, at least among the five molecular species. 

The higher sensitivity of root growth and preferential inhibition of cell proliferation to cell elongation in roots [49] suggested that monoterpenes preferentially inhibited some physiological process related to cell proliferation; because root growth requires both cell proliferation and cell elongation whereas hypocotyl growth only requires elongation of existing cells [50], higher sensitivity of root growth to monoterpenes may be explained by the preferential inhibition of cell proliferation. To examine this possibility and to gain further insight into the mode of action of monoterpenes, Yoshimura *et al.* [40] utilized tobacco (*N. tabacum*) BY-2 suspension-cultured cells as receiver cells. Because BY-2 cells form small cell clusters in which each cells expose most of their surface area to culture media, a synchronous and homogeneous response to any compound applied to culture media is expected [51]. They can either proliferate rapidly or elongate (and accumulate starch) without proliferation, depending on the hormone conditions [52, 53]. Moreover, they can be easily converted to protoplasts, which also, while regenerating cell walls, can proliferate or elongate, depending on the hormonal conditions [54]. Without cell walls, protoplasts are expected to respond more sensitively to the compounds added to the culture media than the cells with intact cell walls. 1,8-Cineole inhibited both proliferation and elongation of the cells in a dose-dependent manner, and the half-maximal inhibitory concentration (*IC_50_*) for cell elongation was lower than that for cell proliferation [40]. Moreover, 1,8-cineole also inhibited starch synthesis with *IC_50 _*value lower than that for cell proliferation. The results clearly demonstrated that the inhibitory effects of 1,8-cineole were not specific to cell proliferation but were rather wide-ranged; 1,8-cineole seemed inhibitory to a variety of physiological activities in the cells. 

We previously suspected that the DNA synthesis, especially that within the organelles, was one of the primary targets of monoterpenes [49], because (i) DNA synthesis in both nuclei and organelles was inhibited in the root apical meristem of monoterpene-treated seedlings [48, 49], (ii) active organelle DNA synthesis and elevation of organelle DNA levels within the cells appeared necessary for subsequent cell propagation [55, 56], (iii) *in vitro* DNA synthesis activity of organelle-nuclei (nucleoids) isolated from BY-2 cells [57] was inhibited by addition of monoterpenes [49]. However, the fact that not only cell proliferation but also cell elongation and starch synthesis were inhibited efficiently by 1,8-cineole ruled out the possibility that the (organelle) DNA synthesis represents the primary target point of monoterpene actions. 

Instead, we hypothesized that deleterious effects of monoterpene on mitochondria might cause disturbances in a wide range of physical and biochemical processes within the target cells. The lipophilic property of monoterpenes, though it is more soluble to water than assumed formerly [38], suggest that they should preferentially accumulate in hydrophobic environment, such as biological membranes, when they entered the target cells. Lipid oxidation and deterioration of membrane integrity in plant cells exposed to monoterpenes [12, 45, 58] suggest that the biological membranes are severely affected by monoterpenes. Lorber and Muller [12] reported a drastic reduction in the number of intact organelles, including mitochondria. A reduction in respiratory oxygen consumption in response to treatment with monoterpenes has been reported in a number of studies using whole plants, dissected organs, and isolated mitochondria [10, 47, 59-61]. Alpha-pinene caused severe reduction of ATP production capacity of mitochondria isolated from maize, which was attributable to uncoupling of oxidative phosphorylation and inhibition of electron transfer [62]. In earlier studies, however, the site of inhibition was suggested to be localized to Krebs cycle [11]. It seems likely that the inhibitory effects of monoterpenes on mitochondria could be expanded to various cellular activities via the reduced ATP production.

In addition to reduced respiration, treatment with monoterpenes causes generation of reactive oxygen species (ROS), oxidative damage to the cells, and induction of antioxidant enzymes (e.g., [45, 63-65]). Production of ROS, and resulting oxidative stress have been proposed as one of the major mechanisms of action of various phytotoxins [66]. As far as we know, the origin of ROS in these monoterpene-treated plants has not conclusively determined, but oxidative burst, generally observed under biotic and abiotic stresses, appears to be the assumed mechanism. In addition, lowered rate of respiratory electron transfer in mitochondria might also result in the formation of ROS through premature release of oxygen before complete reduction. Production of excess ROS, like lowered ATP production by mitochondria, would interfere with various cellular processes, which agrees with the wide range of disturbances observed in BY-2 cells treated with 1,8-cineole.

### VII Role of Cuticular Waxes

We think that the different sensitivity to monoterpenes between roots and hypocotyls, which has been mentioned above, might give us a clue to explore the mechanism of monoterpene actions. As the hypothesis of “preferential inhibition of cell proliferation” has been ruled out, the reason for the different sensitivity still remains to be clarified.

One possible explanation for the different sensitivity between the organs was the difference in the actual concentration of monoterpene around the organs. As shown by C.H. Muller and del Moral [2], monoterpenes are readily adsorbed to containers used for bioassays. Thus, monoterpene concentration in the gas-phase around aerial organs should become lower, while that in the solid/liquid phase around roots should become higher, as the duration of incubation becomes longer. However, this was not the critical factor for the differential inhibition, because root growth was still more sensitive to 1,8-cineole than hypocotyl growth even if the seedlings were grown sandwiched between filter paper wads to maintain the same 1,8-cineole concentrations between roots and hypocotyls [49]. Thus, we hypothesized that the permeability to monoterpenes might be different between the organs. The surface of aerial parts of the plant body is covered with a well-developed cuticule layer while the root surface is covered only poorly [67]. Development of cuticle layer could be indirectly assessed by permeability assay based on staining with toluidine blue (TB) dye [67], and the results of such analysis demonstrated the gradient of permeability (probably negatively correlated to the development of cuticule layer) within the seedlings of various test plants (Fig. (**4**)). While aerial parts (relatively resistant to monoterpenes) were hardly stained with TB (i.e., with little permeability, suggesting presence of well-developed cuticle layer), roots (relatively sensitive to monoterpenes) were stained densely (i.e., with high permeability, indicative of poor development of cuticle layer). Moreover, differential TB staining was also noted within a root. While the root tip region (including root apical meristem where mitosis was effectively inhibited by monoterpenes) was stained heavily, the upper region (corresponding to elongation zone where cell sizes were not influenced by a certain dose of monoterpenes) was stained more faintly. These observations suggest that effectiveness of externally added monoterpenes was negatively correlated to TB permeability (and thus the degree of cuticle development) of the surface of the focal sites. We also found that the seedlings of *Arabidopsis* mutant with deficiency in epicuticular wax synthesis, *yore-yore-1 *[68], exhibited higher sensitivity to 1,8-cineole than those of wild type (unpublished result). 

The observations described above suggest that cuticular layer might act as barrier to the penetration of monoterpenes into the plant body, which seems somewhat different from the earlier view that waxes might be act as a route of monoterpene incorporation [2, 47]. However, observations that the head of glandular hairs, as well as leaf surfaces, of aromatic plants are covered with thick cuticlar layer [3, 30, 69] and that plants treated with monoterpenes exhibit excessive deposit of cuticular waxes [9] suggest that cuticlar layers might indeed act as an “adsorptive barrier” against movement of monoterpenes. Weidenhmer *et al.* [38] reported that solubility of monoterpenes in water was reduced when solid ursolic acid (a natural surfactant) was present, and proposed that the ursolic acid adsorbed the bulk of the monoterpenes added, similar to the action of a solid-phase adsorbent. We propose that the cuticular waxes also act as a potent adsorbent to lipophilic monoterpenes, thereby affect the behavior and distribution of monoterpenes within the microenvironment in and around the target cells. Fischer *et al*. [36] stated that “the major ecological role of cuticlar waxes in allelopathic processes and other biological functions may be due to their fixative property, which enhances long-term retention of active volatiles that would otherwise be lost to volatilization”. We propose that the major physiological role of cuticlar waxes in allelopathic processes involving monoterpenes may be due to their fixative property, which enhances sequestration of the lipophilic but moderately water-soluble compounds that would otherwise dissolve into apoplastic fluid, keeping them away from protoplasm. 

## CONCLUSION

The “*Salvia* phenomenon” is one of the most famous examples of allelopathic interaction between higher plants, for which monoterpenes, especially camphor and 1,8-cineole, have been regarded to be responsible. However, the origin of “bare zone” around the *Salvia* thickets appeared to be not fully attributable to the action of monoterpenes: various factors such as animal activity, phenology of both shrubs and annual herbs, seasonal changes in the environmental conditions, and species-specific response to the monoterpenes, appear to be involved in the formation of the characteristic vegetation patterning. The proposed mechanisms by which *Salvia* monoterpenes affect target plants in the vicinity also include considerable uncertainties. In this short review, we propose that (i) In addition to the initially proposed mechanism (volatilization from living leaf – soil adsorption), leaching (from both living and decomposing plants) and volatilization from decaying litter might contribute, at least during specific seasons. (ii) Volatile monoterpenes can reach target cells even in the absence of continuous “lipophilic corridor”, (iii) The mode of action of monoterpenes produced by *S. leucophylla* may be common, and they are all (more or less) effective to various plant species. (iv) The inhibitory effects of monoterpenes are rather non-specific; they can inhibit a variety of physiological and biochemical processes within the target cells. Such a wide-ranged effect may be explained if primary point of action resides in mitochondrial function or in ROS generation. (v) The cuticular waxes may represent an “adsorptive barrier” against the permeation of monoterpenes from exterior of plant body to the inside of cell wall. Clearly, further analyses are necessary to cralify the mechanism of “*Salvia* phenomenon” and to apply it to human activities such as weed protection in agriculte.

## Figures and Tables

**Fig. (1).**
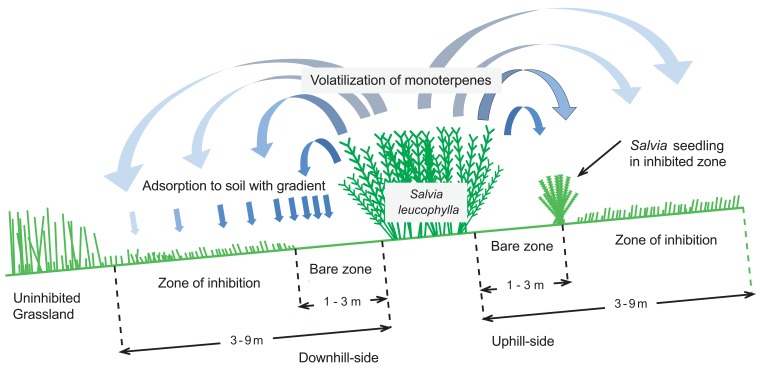
Schematic representation of the “*Salvia* phenomenon”.

**Fig. (2).**
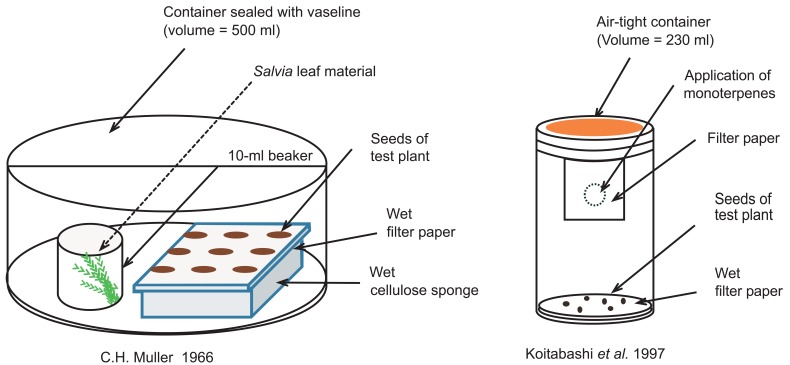
Experimental system of C.H. Muller (1966) and Koitabashi *et al*. (1997).

**Fig. (3).**
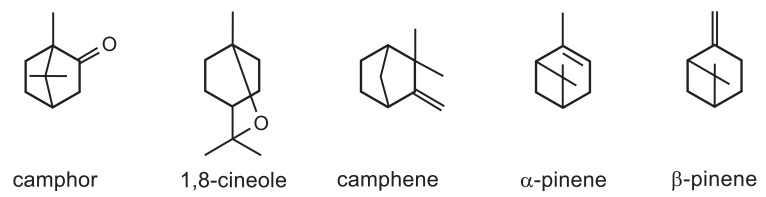
Monoterpenes that W.H. Muller and C.H. Muller found in the *S. leucophylla* leaves.

**Fig. (4).**
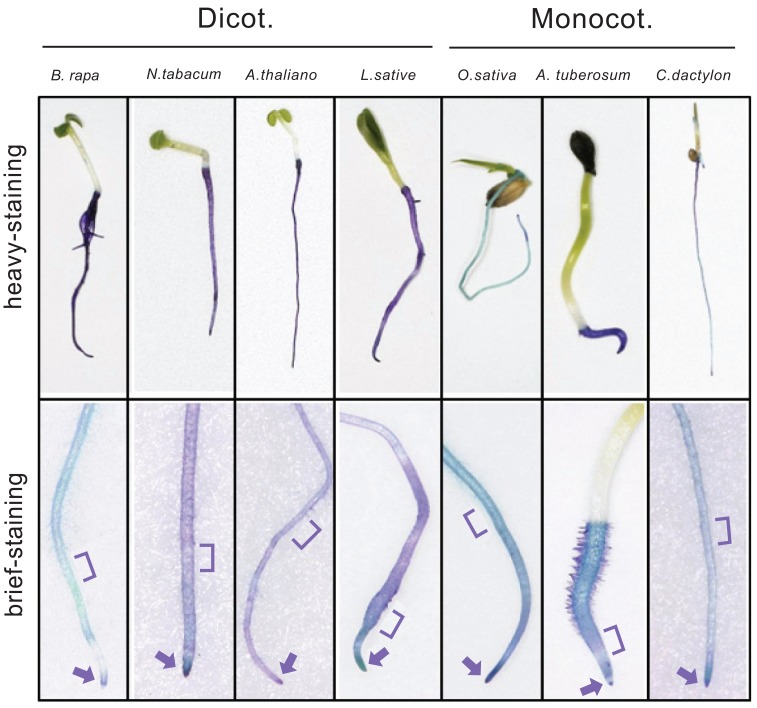
Differences in the permeability (the extent to which cuticular layers develop) as revealed by Toluidine Blue-staining. Arrows, root apical meristem. ], elongation zone.

**Table 1.** IC_50_ Values of the 1,8-cineole for the Inhibition of the Root Growth and Hypocotyl/coleoptile Growth. IC_50_ Values are Expressed in μM in the Atmosphere as Calculated Values, Assuming that the Added 1,8-cineole was Completely Volatilized Within the Container Without Adsorption to Anything in the System

IC_50_ (M) for:	Dicot.				Monocot.		
	*B. rapa*	*N. tabacum*	*A. thaliana*	*L. sativa*	*A. tuberosum*	*O. sativa*	*C. dactylon*
Hypocotyl/coleoptile growth	760	710	840	200	240	720	400
Root growth	150	440	300	140	50	280	190

## References

[1]  Muller CH, Muller WH, Haines BL (1964). Volatile growth inhibitors
produced by aromatic shrubs. Science.

[2]  Muller CH, del Moral R (1966). Soil toxicity induced by terpenes from
*Salvia leucophylla*. Bull. Torrey Bot Club.

[3]  Youngken HW, Heaps W
 (1948). Studies on the leaf of *Salvia leucophylla*. J. Am. Pharm. Assoc.

[4]  Muller CH (1966). The role of chemical inhibition (allelopathy) in vegetational
composition. Bull. Torrey Bot. Club.

[5]  Nilsen ET, Inderjit Mallik AU (2002). Ecological relevance of allelopathy: some considerations
related to Mediterranean, subtropical, temperate, and boreal
forest shrubs. Chemical Ecology of
Plants: Allelopathy in Aquatic and Terrestrial Ecosystems.

[6]  Muller WH, Muller CH (1964). Volatile growth inhibitors produced by
*Salvia* species. Bull. Torrey Bot Club.

[7]  Muller CH (1965). Inhibitory terpenes volatilized from Salvia shrubs. Bull. Torrey Bot. Club.

[8]  Muller WH (1965). Volatile materials produced by *Salvia leucophylla*:
effects on seedling growth and soil bacteria. Botan. Gaz.

[9]  Muller WH, Hauge R (1967). Volatile growth inhibitors produced by
*Salvia leucophylla* effect on seedling anatomy. Bull. Torrey Bot.
Club.

[10]  Muller WH, Lorber P, Haley B (1968). Volatile growth inhibitors
produced by *Salvia leucophylla*: effect on seedling growth and
respiration. Bull. Torrey Bot. Club.

[11]  Muller WH, Lorber P, Haley B, Johnson K (1969). Volatile growth
inhibitors produced by *Salvia leucophylla*: Effect on oxygen uptake
by mitochondrial suspensions. Bull. Torrey Bot Club.

[12]  Lorber P, Muller WH (1976). Volatile growth inhibitors produced by
*Salvia leucophylla*: effects on seedling root tip ultrastructure. Amer. J. Bot.

[13]  Wells PV (1964). Antibiosis as a factor in vegetation patterns. Science.

[14]  Muller CH, Muller WH (1964). Response to P.V. Wells (Antibiosis as
a factor in vegetation patterns). Science.

[15]  Bartholomew B (1970). Bare zone between California shrub and grassland
communities: the role of animals. Science.

[16]  Muller CH, del Moral R (1971). Role of animals in suppression of
herbs by shrubs. Science.

[17]  Bartholomew B (1971). Response to C.H. Muller and R. del Moral (Role
of animals in suppression of herbs by shrubs). Science.

[18]  Halsey RW (2004). In search of allelopathy: an eco-histrical view of the
investigation of chemical inhibition in California coastal sage
scrub and chamise chaparral. J. Torrey Bot. Soc.

[19]  Halligan JP (1973). Bare areas associated with shrub standsin grassland:
the case of Artemisia californica. BioScience.

[20] Emboden WA, Lewis H (1967). Terpenesas toxonomiccharactersin
*Salvia* section Audibertia. Brittonia.

[21]  Mancini E, Arnold NA, De Martino L, De Feo V, Formisano C, Rigano D, Senatore F (2009). Chemical composition and phytotoxic
effects of essential oils of *Salvia hierosolymitana* Boiss. and *Salvia
multicaulis* Vahl. var. *simplicifolia Boiss*, growing wild in Lebanon. Molecules.

[22]  De Martino L, Roscigno, Mancini E, De Falco E, De Feo 
V (2010). Chemical composition and antigerminative activity of the essential
oils from five *Salvia* species. Molecules.

[23]  Epling C (1938). The Californian Salvias. A review of *Salvia*, section
*Audibertia*. Annals of the Missouri Botanical Garden.

[24]  Degenhardt J, Kollner TG, Gershenzon J (2009). Monoterpene and
sesquiterpene synthases and origin of terpene skeltal diversity in
plants. Phytochemistry.

[25]  Fahnrich A, Krause K, Piechulla B (2011). Product variability of the
'cineole cassette' monoterpene synthases of related *Nicotiana* species. Mol. Plant.

[26]  Wise ML, Savage TJ, Katahira E, Croteau RB (1998). Monoterpene
synthases from common sage (*Salvia officinales*). J. Biol. Chem.

[27]  Croteau R, Alonso WR, Koepp AE, Johnson MA (1994). Biosynthesis
of monoterpenes: partial purification, characterization, and
mechanism of action of 1,8-cineole synthase. Arch. Biochem. Biophys.

[28]  Kampranis SC, Loannidis D, Purvis A, Mahrez W, Ninga E, 
Katerelos NA, Anssour S, Dunwell JM, Degenhardt J, 
Makris AM, Goodenough PW, Johonson CB (2007). Rational conversion
of substrate and product specificity in a *Salvia* monoterpene
synthase: structural insights into the evolution of terpene synthase
function. Plant Cell.

[29]  Chen F, Ro D-K, Petri J, Gershenzon J, Bohlmann J, Pichersky 
E, Tholl D (2004). Characterization of a root-specific Arabidopsis
terpene synthase responsible for the formation of the monoterpene
1,8-cineole. Plant Physiol.

[30]  Serrato-Valenti G, Bisio A, Cornara L, Ciarallo G (1997). Structural
and histochemical investigation of the glandular trichomes of *Salvia
aurea* L. leaves, and chemical analysis of the essential oil. Annals
of Botany.

[31]  Duke SO (1994). Glandular trichomes - a focal point of chemical and
structural interactions. Int. J. Plant Sci.

[32]  Croteau R, Felton M, Karp F, Kjonaas R (1981). Relationship of
camphor biosynthesis to leaf development in sage (*Salvia officinalis*). Plant Physiol.

[33]  Tyson BJ, Dement WA, Mooney HA (1974). Volatilisation of terpenes
from *Salvia mellifera*. Nature.

[34]  Dement WA, Tyson BJ, Mooney HA (1975). Mechanism of
monoterpene volatilization in *Salvia mellifera*. Phytochemistry.

[35]  Rasmussen RA, Went FW (1965). Volatile organic material of plant
origin in the atmosphere. Proc. Natl. Acad. Sci. U.S.A.

[36]  Fischer NH, Williamson GB, Weidenhamer JD, Richardson DR (1994). In search of allelopathy in the Florida scrub: the role of terpenoids. J. Chem. Ecol.

[37]  Fischer NH, Tanrisever N, Williamson GB, Cutler H (1988). Allelopathy in the
Florida scrub community as a model for natural herbicide actions. Natural products: potential in Agriculture.

[38]  Weidenhamer JD, Macias FA, Fischer NH, Williamson GB (1993). Just how insoluble are monoterpenes?. J. Chem. Ecol.

[39] Fischer NH, Tomas-Barberan FA, Harborne JB (1991). Plant terpenoids as allelopathic agents. Ecological Chemistry and Biochemistry
of Plant Terpenenoids.

[40]  Yoshimura H, Sawai Y, Tamotsu S, Sakai A (2011). 1,8-Cineole
inhibits both proliferation and elongation of BY-2 cultured tobacco
cells. J. Chem. Ecol.

[41]  Duke SO, Oliva A, Macias FA, Galindo JCG, Molinillo JMG, Cutler HG (2004). Mode of action of phytotoxic terpenoids. Allelopathy, Chemistry and Mode Of Action Of Allelochemicals.

[42]  Dayan FE, Romagni JG, Duke SO (2000). Investigation of the mode
of action of natural phytotoxins. J. Chem. Ecol.

[43]  Romagni JG, Allen SN, Dayan FE (2000). Allelopathic effects of
volatile cineoles on two weedy plant species. J. Chem Ecol.

[44]  Schulz M, Kussmann P, Knop M, Kriegs B, Gresens F, 
Eichert T, Ulbrich A, Marx F, Fabricius H, Goldbach H, 
Noga G (2007). Allelopathic monoterpenes interfere with *Arabidopsis
thaliana* cuticular waxes and enhance transpiration. Plant Signal.
Behav.

[45]  Zunino MP, Zygadlo JA (2004). Effects of monoterpenes on lipid
oxidation in maize. Planta.

[46]  Chaimovitsh D, Abu-Abied M, Belausov E, Rubin B, Dudai 
N, Sadot E (2010). Microtubules are an intracellular target of the plant
terpene citral. Plant J.

[47]  Abrahim D, Braguini L, Kelmer-Bracht MK, Ishii-Iwamoto 
EL (2000). Effects of four monoterpenes on germination, primary root
growth and mitochondrial respiration of maize. J. Chem. Ecol.

[48]  Koitabashi R, Suzuki T, Kawazu T, Sakai A, Kuroiwa H, 
Kuroiwa T (1997). 1 8-cineole inhibits root growth and DNA synthesis in
the root apical meristem of *Brassica campestris* L. J. Plant Res.

[49]  Nishida N, Tamotsu S, Nagata N, Saito C, Sakai A (2005). Allelopathic
effects of volatile monoterpenoids produced by *Salvia leucophylla*
inhibition of cell proliferation and DNA synthesis in the
root apical meristem of *Brassica campestris* seedlings. J. Chem.
Ecol.

[50]  Obroucheva NV (1999). Seed Germination: A Guide to the Early Stages.

[51]  Sakai A, Miyazawa Y, Kuroiwa T, Nagata T, Hasezawa S, Inze D (2004). Studies on dynamic changes
of organelles using tobacco BY-2 as the model plant cell line. Tobacco BY-2 cells. Biotechnology
in Agriculure and Forestry.

[52]  Nagata T, Nemoto Y, Hasezawa S (1992). Tobacco BY-2 cell line as
the 'Hella” cell in the cell biology of higher plants. Int. Rev. Cytol.

[53]  Sakai A, Yashiro K, Kawano S, Kuroiwa T (1996). Amyloplast formation
in cultured tobacco cells effects of plant hormones on multiplication
size, and starch content. Plant Cell Rep.

[54]  Hasezawa S, Syono K (1983). Hormonal control of elongation of tobacco
cells derived from protoplasts. Plant Cell Physiol.

[55]  Suzuki T, Kawano S, Sakai A, Fujie M, Kuroiwa H, Nakamura 
H, Kuroiwa T (1992). Preferential mitochondrial and plastid DNA
synthesis before multiple cell divisions in *Nicotiana tabacum*. J.
Cell Sci.

[56]  Suzuki T, Sakai A, Kawano S, Kuroiwa T (1996). Organelle DNA
synthesis before cell nuclear replication is essential for subsequent
cell propagation. Cytologia.

[57]  Sakai A, Suzuki T, Nagata N, Sasaki N, Miyazawa Y, Saito 
C, Inada N, Nishimura Y, Kuroiwa T (1999). Comparative analysis of
DNA synthesis activity in plastid-nuclei and mitochondrial-nuclei
simultaneously isolated from cultured tobacco cells. Plant Sci.

[58]  Fischer NH, Putnam A.R, Tang C.-S (1986). The function of mono and sesquiterpenes as plant
germination and growth regulators. The Science of Allelopathy.

[59]  Peñuelas J, Ribas-Carbo M, Giles L (1996). Effects of allelochemicals
on plant respiration and oxygen isotope discrimination by the alternative
oxydase. J. Chem. Ecol.

[60]  Abrahim D, Takahashi L, Kelmer-Bracht AM, Ishii-Iwamoto 
EL (2003a). Effects of phenolic acids and monoterpenes on the mitochondrial
respiration of soybean hypocotyl axes. Allelopathy J.

[61]  Abrahim D, Fransischini A C, Pergo EM, Kelmer-Bracht 
AM, Ishii-Iwamoto EL (2003b). Effects of a-pineneon the mitochondrial
respiration of maize seedlings. Plant Physiol. Biochem.

[62]  Ishii-Iwamoto EL, Abrahim D, Sert MA, Bonato CM, Kelmer-Bracht AM, Bracht A, Reigosa MJ, Pedrol N, González L (2006). Mitochondria as a site of allelochemical action. Allelopathy: A Physiological Process with Ecological Implication.

[63]  Singh HP, Batish DR, Kaur S, Arora K, Kohli (2006). ??-pinene
inhibits growth and induces oxidative stress in roots. Ann. Bot.

[64]  Singh HP, Kaur S, Mittal S, Batish DR, Kohli RK (2009). Essential
oil of *Artemisia scoparia* inhibits plant growth by generating
reactive oxygen species and causing oxidative damage. J. Chem.
Ecol.

[65]  Chowhan N, Singh HP, Batish DR, Kohli RK (2011). Phytotoxic
effects of ??-pinene on early growth and associated biochemical
changes in rice. Acta. Physiol. Plant.

[66]  Weir TL, Park S-W, Vivanco JM (2004). Biochemical and physiological
mechanisms mediated by allelochemicals. Curr. Opin
Plant Biol.

[67]  Bessire M, Chassot C, Jacquat A-C, Humphry M, Borel 
S, etétot JM-C, Métraux J-P, Nawrath C (2007). A permeable cuticle
in Arabidopsis leads to a strong resistance to *Botrytis cinera*. EMBO J.

[68]  Kurata T, Kawabata-Awai C, Sakuradani E, Shimizu S, 
Okada K, Wada T (2003). The *YORE-YORE* gene regulates multiple aspects
of epidermal cell differentiation in Arabidopsis. Plant J.

[69]  Turner GW, Gershenzon, Croteau RB (2000). Development of peltate
glandular trichomes of peppermint. Plant Physiol.

